# Convolutional neural network-based automatic heart segmentation and quantitation in ^123^I-metaiodobenzylguanidine SPECT imaging

**DOI:** 10.1186/s13550-021-00847-x

**Published:** 2021-10-12

**Authors:** Shintaro Saito, Kenichi Nakajima, Lars Edenbrandt, Olof Enqvist, Johannes Ulén, Seigo Kinuya

**Affiliations:** 1 Department of Nuclear Medicine, Kanazawa University Graduate School of Medicine, 13-1 Takara-machi, Kanazawa, 920-8640 Japan; 2 Department of Functional Imaging and Artificial Intelligence, Kanazawa University Graduate School of Medicine, 13-1 Takara-machi, Kanazawa, 920-8640 Japan; 3 Department of Clinical Physiology, Region Västra Götaland, Sahlgrenska University Hospital, Gothenburg, Sweden; 4 Department of Chalmers, University of Technology, Gothenburg, Sweden; 5 Eigenvision, Malmö, Sweden; 6 Department of Nuclear Medicine, Kanazawa University, Kanazawa, Japan

**Keywords:** Artificial intelligence, Myocardial sympathetic imaging, Innervation, Heart-to-mediastinum ratio, Washout rate

## Abstract

**Background:**

Since three-dimensional segmentation of cardiac region in ^123^I-metaiodobenzylguanidine (MIBG) study has not been established, this study aimed to achieve organ segmentation using a convolutional neural network (CNN) with ^123^I-MIBG single photon emission computed tomography (SPECT) imaging, to calculate heart counts and washout rates (WR) automatically and to compare with conventional quantitation based on planar imaging.

**Methods:**

We assessed 48 patients (aged 68.4 ± 11.7 years) with heart and neurological diseases, including chronic heart failure, dementia with Lewy bodies, and Parkinson's disease. All patients were assessed by early and late ^123^I-MIBG planar and SPECT imaging. The CNN was initially trained to individually segment the lungs and liver on early and late SPECT images. The segmentation masks were aligned, and then, the CNN was trained to directly segment the heart, and all models were evaluated using fourfold cross-validation. The CNN-based average heart counts and WR were calculated and compared with those determined using planar parameters. The CNN-based SPECT and conventional planar heart counts were corrected by physical time decay, injected dose of ^123^I-MIBG, and body weight. We also divided WR into normal and abnormal groups from linear regression lines determined by the relationship between planar WR and CNN-based WR and then analyzed agreement between them.

**Results:**

The CNN segmented the cardiac region in patients with normal and reduced uptake. The CNN-based SPECT heart counts significantly correlated with conventional planar heart counts with and without background correction and a planar heart-to-mediastinum ratio (*R*^2^ = 0.862, 0.827, and 0.729, *p* < 0.0001, respectively). The CNN-based and planar WRs also correlated with and without background correction and WR based on heart-to-mediastinum ratios of *R*^2^ = 0.584, 0.568 and 0.507, respectively (*p* < 0.0001). Contingency table findings of high and low WR (cutoffs: 34% and 30% for planar and SPECT studies, respectively) showed 87.2% agreement between CNN-based and planar methods.

**Conclusions:**

The CNN could create segmentation from SPECT images, and average heart counts and WR were reliably calculated three-dimensionally, which might be a novel approach to quantifying SPECT images of innervation.

**Supplementary Information:**

The online version contains supplementary material available at 10.1186/s13550-021-00847-x.

## Introduction

Estimating sympathetic nervous activity using ^123^I-metaiodobenzylguanidine (MIBG) is a valuable adjunct for assessing the severity, prognosis, and effects of treatment for heart failure, arrhythmogenic disease, and neurological diseases such as dementia with Lewy bodies and Parkinson's disease [1–8].

The heart-to-mediastinum ratio (HMR) and washout rate (WR) in planar images are common indicators of sympathetic nervous activity [9]. Some studies have shown good reproducibility using ^123^I-MIBG planar images [9–11]. However, depending on the method of regions of interest (ROI) definition, up to about 40% of results might located lying in a gray zone around the cut-off, through which normal and abnormal innervation are differentiated in the clinical context [12]. In Japan, the HMR and WR have been calculated from planar images using smartMIBG, a semiautomated ROI setting software developed under collaboration with FUJIFILM Toyama Chemical Co. Ltd., Tokyo, Japan [9], whereas ROI has also been set manually according to American Society of Nuclear Cardiology and European recommendations [13–15].

Single-photon emission computed tomography (SPECT) generates three-dimensional (3D) images that are potentially useful to discriminate organ and background activities that overlap the heart. Degrees of segmental defects can also be scored using the 17-segment model applied in myocardial perfusion imaging (MPI) [1]. However, 3D ^123^I -MIBG distribution seemed to be heterogeneous based on SPECT studies [16]. Besides, segmental uptake differs among ^123^I-MIBG SPECT images of individuals. The normal database for ^123^I-MIBG sympathetic imaging shows relatively decreased activity in the inferior wall, and this was more prominent in late images [17]. To set three-dimensional ROI using the conventional method is difficult in practice.

Here, we present an artificial intelligence (AI) method based on convolution neural networks (CNNs) to define cardiac lesions and calculate heart counts without a manual setting. Deep learning algorithms, in particular CNNs, have become the methodology of choice for analyzing medical images [18]. The deep learning approach has been applied to assess conditions such as cardiovascular diseases and prostate cancer using radiology and nuclear medicine [19, 20]. The CNN can directly identify patterns in 3D SPECT images, which allows the classification of each pixel into anatomical components in the image. However, 3D CNN segmentation and automatic calculation of heart counts for ^123^I-MIBG SPECT have not been reported because cardiac uptake is quite variable and sometimes significantly reduced in patients with severe heart failure and dementia with Lewy bodies.

The present study aimed to create a segmentation method and to calculate heart counts and WR in ^123^I-MIBG SPECT images using CNN. We also compared this novel approach with conventional quantitation based on planar images.

## Methods

### Patients

We assessed 51 consecutive patients with heart and neurological diseases by ^123^I-MIBG planar and SPECT imaging at Kanazawa University Hospital during 2018 and 2019. We selected data from 48 patients with visible lung and liver uptake to evaluate standard organ segmentation of ^123^I-MIBG images. One patient had low accumulation in the liver parenchyma due to a giant liver cyst, and two others had low accumulation in the lungs partly due to leakage at antecubital injection sites. Table 1 shows the characteristics of the 48 patients (male, *n* = 32; female, *n* = 16; average age, 68.4 ± 11.7; range, 26–84 years; weight, 61.1 ± 13.5; range, 28.8–101 kg; body mass index, 23.0 ± 4.1; range, 16–33). Neurological diseases in 27 patients comprised Parkinson's disease (*n* = 4), dementia with Lewy bodies (*n* = 2), familial amyloid polyneuropathy (*n* = 6), and other neurological diseases including progressive supranuclear palsy and related movement disorders (*n* = 15). Heart diseases in 21 patients comprised chronic heart failure (*n* = 13), arrhythmia (*n* = 5), and cardiomyopathy (*n* = 3). Cardiac ^123^I-MIBG uptake was considerably reduced to HMR of < 1.5 in 17 patients. The left ventricular ejection fraction (EF) measured by echocardiography (*n* = 38) was 56.1% ± 17.3% (24–77%), whereas EF was not available in 10 patients with neurological diseases.

**Table 1:** Clinical characteristics of the patients

	Patients (*n* = 48)
Male	32 (67)
Age (years)	68.4 ± 11.7
Body weight (kg)	61.1 ± 13.5
Body mass index (kg/m^2^)	23.0 ± 4.1
Neurological diseases	27 (56)
Parkinson's disease	4
Dementia with Lewy bodies	2
Familial amyloid polyneuropathy	6
Other neurological diseases including progressive supranuclear palsy	15 (44)
Heart diseases	21
Chronic heart failure	13
Arrhythmia	5
Cardiomyopathy	3
Reduced cardiac uptake	17 (35)
Left ventricular ejection fraction	56.1 ± 17.3

Data are shown as *n*, *n* (%), means ± standard deviation unless otherwise indicated.

### ^123^I-MIBG imaging

Anterior planar and SPECT images were acquired using an Anger camera (Siemens Healthcare, Tokyo, Japan) equipped with a low-medium-energy (LME) collimator from 15–20 (early phase) and 180–240 (late phase) min after the patients received an intravenous injection of ^123^I-MIBG (111 MBq, FUJIFILM Toyama Chemical Co. Ltd., Tokyo, Japan). The ^123^I energy was centered at 159 keV with a window of 15% or 20%.

Planar images were acquired for 5 min under conditions of a 256 × 256 matrix, 2.4-mm pixels, and zoom factor 1.0, and SPECT images were acquired for 30 s per view under conditions of a 64 × 64 matrix, 6.6-mm pixels, zoom factor 1.45, 60 projections, 360° circular orbit (radius of rotation 24 cm), and rotation radius 24 cm. The SPECT data were reconstructed using filtered back projection (FBP).

### Planar image analysis

Early (E) and late (L) average heart counts in planar images (planar H_E_ and H_L_, unit counts/pixel) and average mediastinal counts (planar M_E_ and M_L_, unit counts/pixel) were calculated using semiautomated smartMIBG software to set ROI as described in detail elsewhere [9]. In brief, the software algorithm uses a circular heart ROI and a mediastinal ROI that was 10% of the width of the body and a 30% of the height of the mediastinum. After pointing into the center of the heart, all processing is automated, and manual modifications can be added as required.

Early and late heart counts in planar images were calculated using the following formulae for planar H_BC_, planar H, and planar HMR.

Planar H_BC_ and planar H were divided by a decay correction factor (DCF) and injected dose (MBq)/ kg body weight (BW). The DCF was calculated as 0.5^ (time [h] between early and late imaging/13). If the interval between early and late was 3 h, the DCF was 0.85. The timing of early imaging was then set at zero (namely DCF = 1).

$${\text{Planar}}\,{\text{H}}_{{{\text{BC}}}} ,\,{\text{with}}\,{\text{background}}\,{\text{correction}}\,\left( {{\text{BGC}}} \right) \, = {\text{ (Planar}}\,{\text{H - M)}}/{\text{DCF}}/({\text{injected}}\,{\text{dose}}/{\text{kg}}\,{\text{BW}}),$$

$${\text{Planar}}\,{\text{H}},\,{\text{without}}\,{\text{BGC}} = {\text{Planar}}\,{\text{H}}/{\text{DCF}}/({\text{injected}}\,{\text{dose}}/{\text{kg}}\,{\text{BW}}),$$

$${\text{Planar}}\,{\text{HMR}} = {\text{Planar}}\,{\text{H}}/{\text{Planar}}\,{\text{M}}.$$

Washout rates (WR, %) were calculated using the following formulae for planar WR_BC_, planar WR_NC_, and planar WR_HMR_ as:

$${\text{Planar}}\,{\text{WR}}_{{{\text{BC}}}} ,\,{\text{with}}\,{\text{background}}\,{\text{correction}}\,\left( {{\text{BGC}}} \right) = [({\text{Planar}}\,{\text{H}}_{{\text{E}}} - {\text{M}}_{{\text{E}}} ) - ({\text{Planar}}\,{\text{H}}_{{\text{L}}} - {\text{M}}_{{\text{L}}} )/{\text{DCF}}]/({\text{Planar}}\,{\text{H}}_{{\text{E}}} - {\text{M}}_{{\text{E}}} ) \times \, 100$$

$${\text{Planar}}\,{\text{WR}}_{{{\text{NC}}}} \,{\text{without}}\,{\text{BGC}} = \left( {{\text{Planar}}\,{\text{H}}_{{\text{E}}} - {\text{ Planar}}\,{\text{H}}_{{\text{L}}} /{\text{DCF}}} \right)/{\text{Planar}}\,{\text{H}}_{{\text{E}}} \times 100$$

$${\text{Planar}}\,{\text{WR}}_{{{\text{HMR}}}} = {\text{ (Planar}}\,{\text{H}}_{{\text{E}}} /{\text{M}}_{{\text{E}}} - {\text{Planar}}\,{\text{H}}_{{\text{L}}} /{\text{M}}_{{\text{L}}} {)}/({\text{Planar}}\,{\text{H}}_{{\text{E}}} /{\text{M}}_{{\text{E}}} ) \, \times 100$$

### Segmentation based on CNN

We used the following two-step model:

1. Early and late images were registered using uptake in the liver and lungs that is highly visible in both images.
2. The heart was directly segmented using both images as input and a single volume as output. All models were trained and evaluated using fourfold cross-validation.

### Registration

**Fig. 1:**
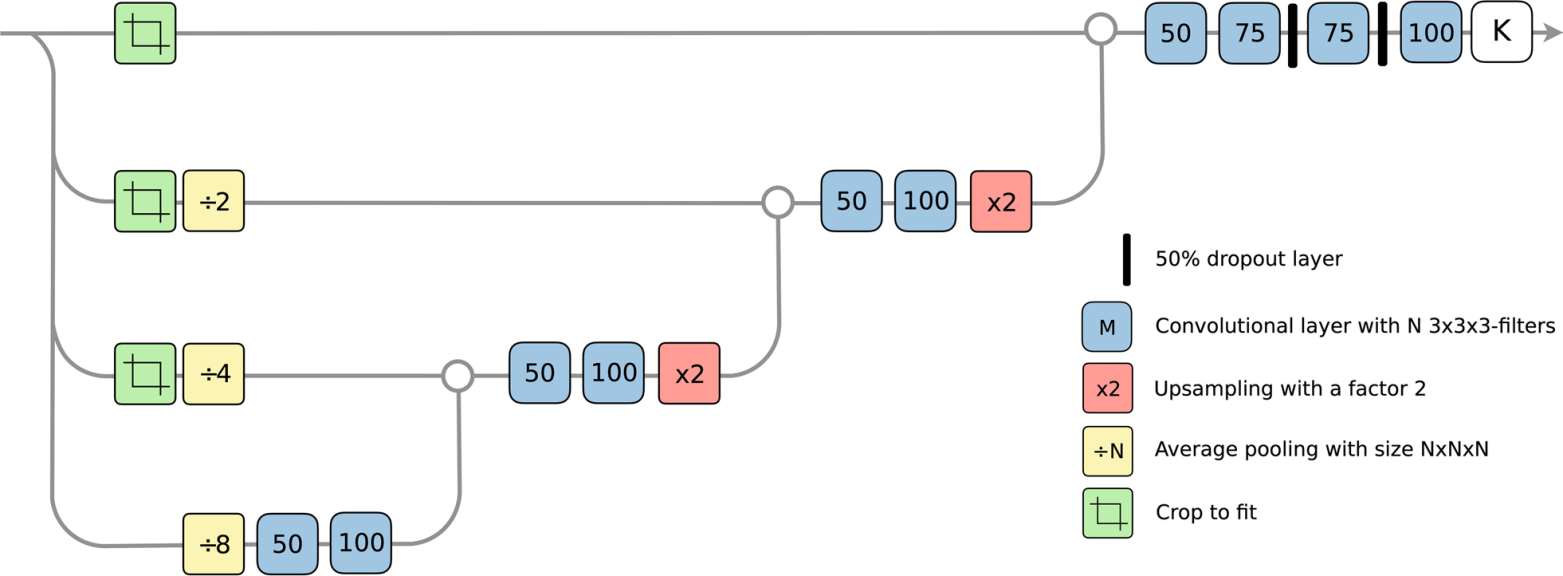
Architecture of CNN used to segment lungs and liver. Convolution layers do not use padding. Input shape to network is 72 × 72 × 72 pixel cube; output shape is 8 × 8 × 8 pixel cube.

We trained the CNN to segment the lungs and liver on early and late SPECT images using ADAM [21] and a negative log-likelihood loss with an initial learning rate of 0.001. Images in each cross-validation fold were divided 80%/20% into training and validation sets, respectively, using the CNN architecture described in Fig. 1. The batch size was 150 and the model stopped training when the validation loss remained stable for 10 epochs. The resulting segmentations were converted to binary masks and used to register early and late images with Elastix [22]. The advanced mean square metric was used with the full image sampler and 200 iterations of gradient descent.

### Heart segmentation

To create a target segmentation for a given early and late image pair, manual heart segmentation masks were aligned using the transformation computed above. The result was fractional labeling with heart probabilities between 0 and 1 depending on whether or not the two aligned segmentations agreed. Using this target volume, the CNN was trained taking the two aligned SPECT volumes as input. We used the same training pipeline as described [23], but to avoid excluding uptake from the heart due to under-segmentation, the background loss was set to 0 for all pixels within 1.2 cm (2 pixels) from the heart that did not overlap with lungs or the liver in either of the aligned masks.

### CNN-based calculation of average heart counts and WR

We calculated SPECT early and late average heart counts per pixel (Early H_CNN_ and Late H_CNN_) and SPECT washout rate (WR_CNN_) using CNN-based heart segmentation. The SPECT H_CNN_ and WR_CNN_ were determined by taking the average counts in the heart VOI from early and late images without background or reference volumes. The SPECT H_CNN_ and WR_CNN_ were calculated as:

$${\text{SPECT}}\,{\text{H}}_{{{\text{CNN}}}} \,{\text{without}}\,{\text{BGC}} = {\text{H}}_{{{\text{CNN}}}} /{\text{DCF}}/({\text{Injected}}\,{\text{dose}}/{\text{kg}}\,{\text{BW}})$$

$${\text{SPECT}}\,{\text{WR}}_{{{\text{CNN}}}} \,{\text{without}}\,{\text{BGC }} = ({\text{Early}}\,{\text{H}}_{{{\text{CNN}}}} - {\text{Late}}\,{\text{H}}_{{{\text{CNN}}}} /{\text{DCF}})/{\text{Early}}\,{\text{H}}_{{{\text{CNN}}}} \times \, 100$$

### Comparison of CNN-based and conventional quantitation

We investigated correlations between SPECT H_CNN_ and planar H_BC_, planar H, and planar HMR for each early and late image. We also investigated correlations between SPECT WR_CNN_ and planar WR_BC_, planar WR_NC_, and planar WR_HMR_. Cutoff values for planar WR parameters to distinguish normal from abnormal determined from standard values created using JSNM working group databases (*n* = 62) were: planar WR_BC_ 34.0%, planar WR_NC_ 30.1%, planar WR_HMR_ = 14.2% [24]. The cutoff for SPECT WR_CNN_ was determined from linear regression lines determined by the relationship between planar and SPECT WR. We divided images into normal and abnormal groups, according to the cutoff values for SPECT WR_CNN_ and planar WR parameters, and then analyzed agreement between them.

### Statistical analysis

Data are expressed as means and standard deviation (SD). Differences in average heart counts and WR between SPECT and planar images regarding were analyzed using *t* tests and two-way analysis of variance. Differences among WR were also analyzed by Bland–Altman plot [25]. Relationships between SPECT and planar methods were assessed by linear regression analysis. Agreement between automated and manual segmentations was estimated using the Sørensen–Dice (Dice) index as numbers of overlapping voxels. All data were statistically analyzed using JMP version 14 (SAS Institute Inc., Cary, NC, USA). Values with *p* ≤ 0.05 were considered statistically significant.

## Results

### Segmentation on images using CNN

**Fig. 2:**
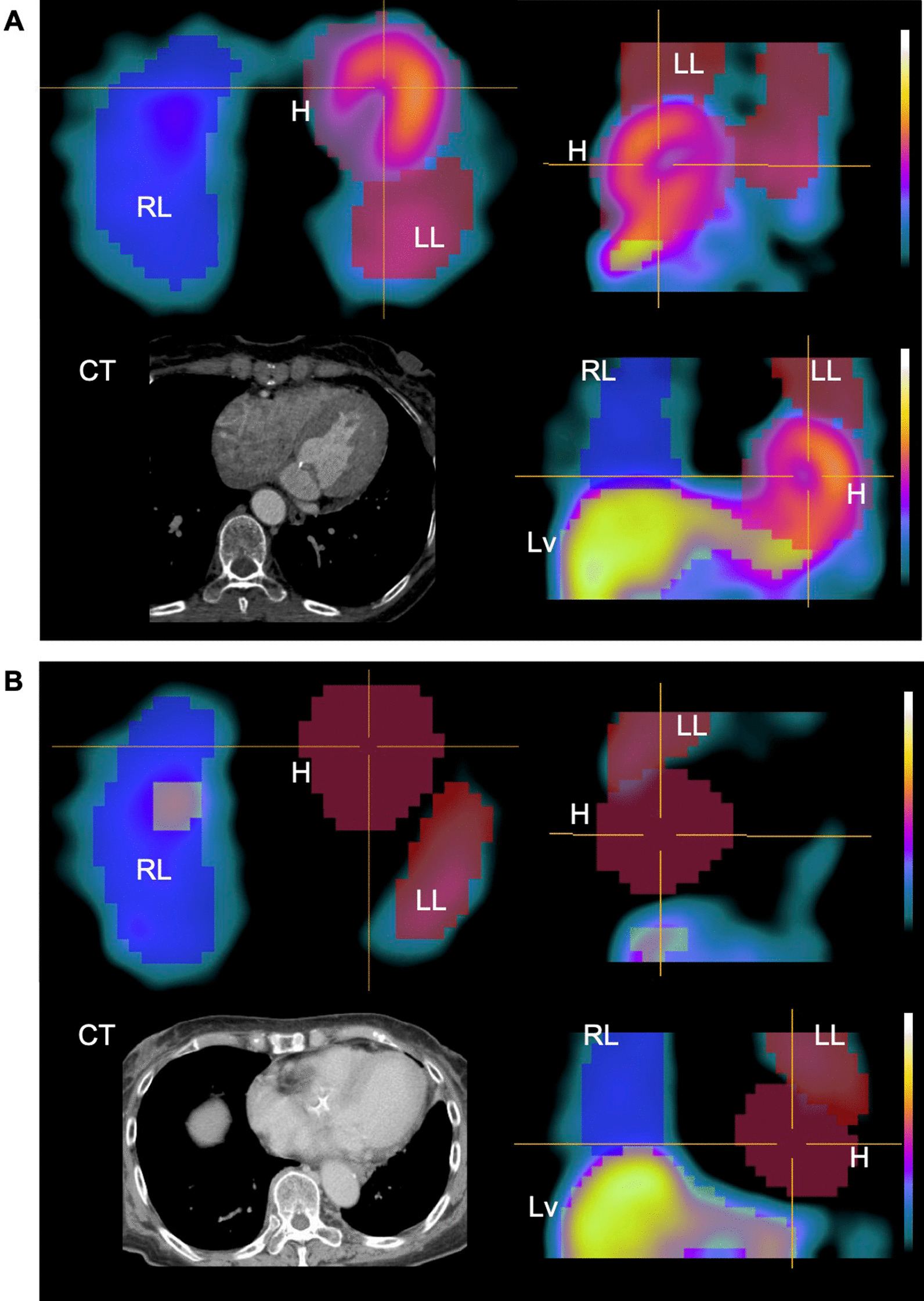
CNN-based segmentation images with ^123^I-MIBG SPECT data. Patients with normal (**A**) and reduced (**B**) uptake. Heart segmentation is correctly identified without anatomical CT images. Liver and lungs are naturally segmented as original organs. Contrast-enhanced X-ray CT images, which were performed for different purposes, are shown as an anatomical reference. H, heart; LL, left lung; Lv, liver; RL, right lung.

Figure 2 shows examples of CNN-based segmentation. The CNN method correctly identified cardiac regions in patients with normal and reduced uptake. Additionally, the heart, liver, and lungs were appropriately segmented in a natural anatomical form as the original organs. The CNN method did not generate sub-diaphragmatic artifacts, and liver and heart segmentation did not overlap in any patients. However, the CNN did not appropriately segment these organs due to high accumulation in an expanding renal pelvis in one patient, and these data were excluded from further statistical analysis. The automatic segmentation had a Sørensen–Dice (Dice) index for early and late SPECT images of 0.63 ± 0.15, recall of 0.82 ± 0.15, and precision of 0.54 ± 0.19.

### *SPECT H*_*CNN*_* versus planar H*_*BC*_*, planar H, and planar HMR*

**Fig. 3:**
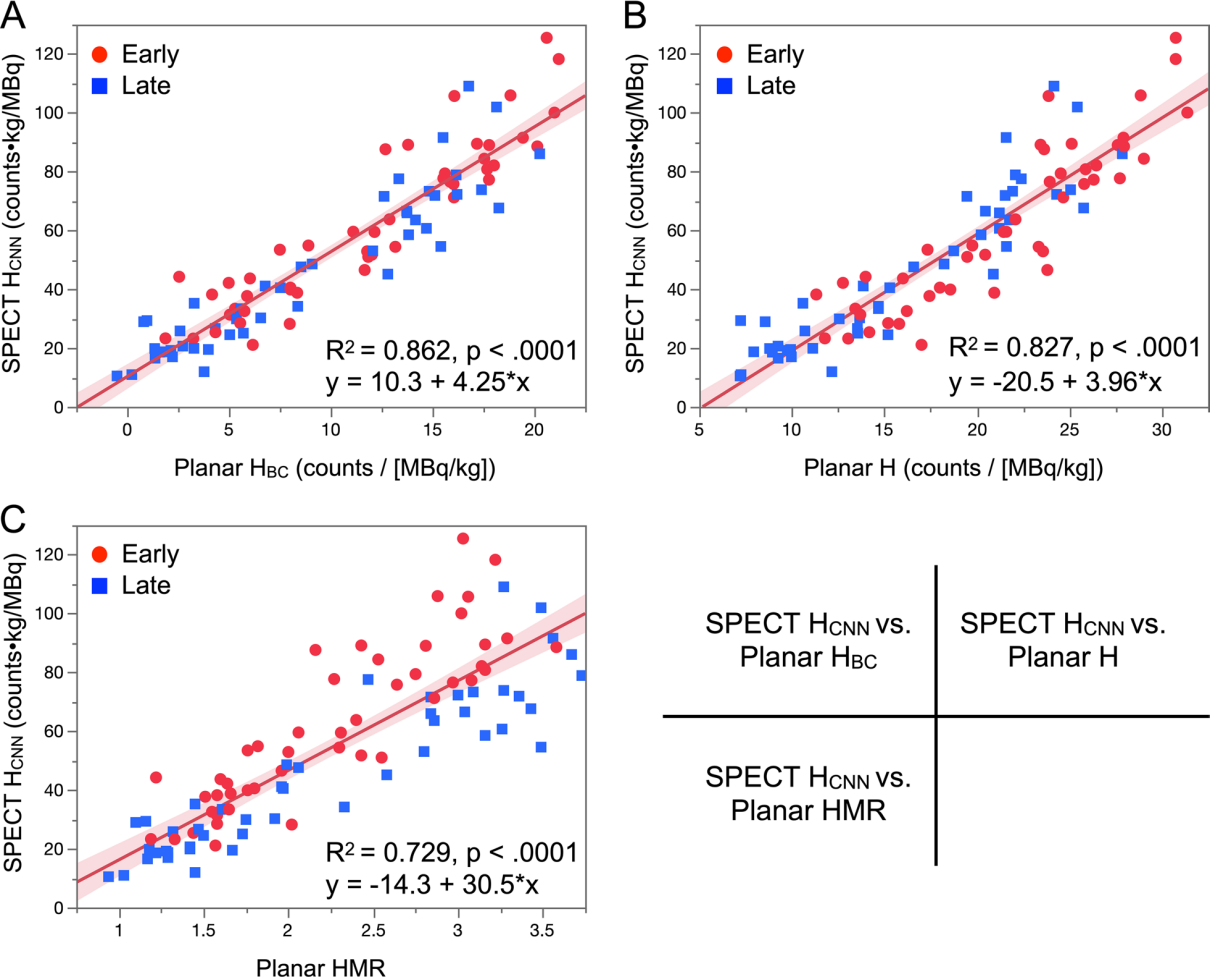
Relationship of average heart counts calculated from SPECT images using CNN and from conventional early and late planar images. SPECT H_CNN_ vs. planar H_BC_ (**A**), planar H (**B**), and planar HMR (**C**). Red circles and blue squares, early and late images, respectively. Shaded area, confidence of fit.

The average heart counts were compared between SPECT images using CNN and planar images using the conventional method for early and late imaging. The correlation between SPECT H_CNN_ and planar H_BC_ with background correction was close (SPECT H_CNN_ = 10.3 + 4.25 × planar H_BC_; *R*^2^ = 0.862, *p* < 0.0001; Fig. 3A). Correlations were also good between SPECT H_CNN_ and planar H without background correction, and between SPECT H_CNN_ and planar HMR (*R*^2^ = 0.827 and 0.729, *p* < 0.0001, respectively; Fig. 3B and C). Correlations were positive between SPECT H_CNN_ and the planar parameters H_BC_, H, and HMR even in patients with reduced myocardial ^123^I-MIBG uptake with HMR < 1.5, (*R*^2^ = 0.460–0.498, *p* < 0.0001 for all; Additional file 1: Figure S1).

### *SPECT WR*_*CNN*_* versus planar WR*_*BC*_*, planar WR*_*NC*_*, and planar WR*_*HMR*_

**Fig. 4:**
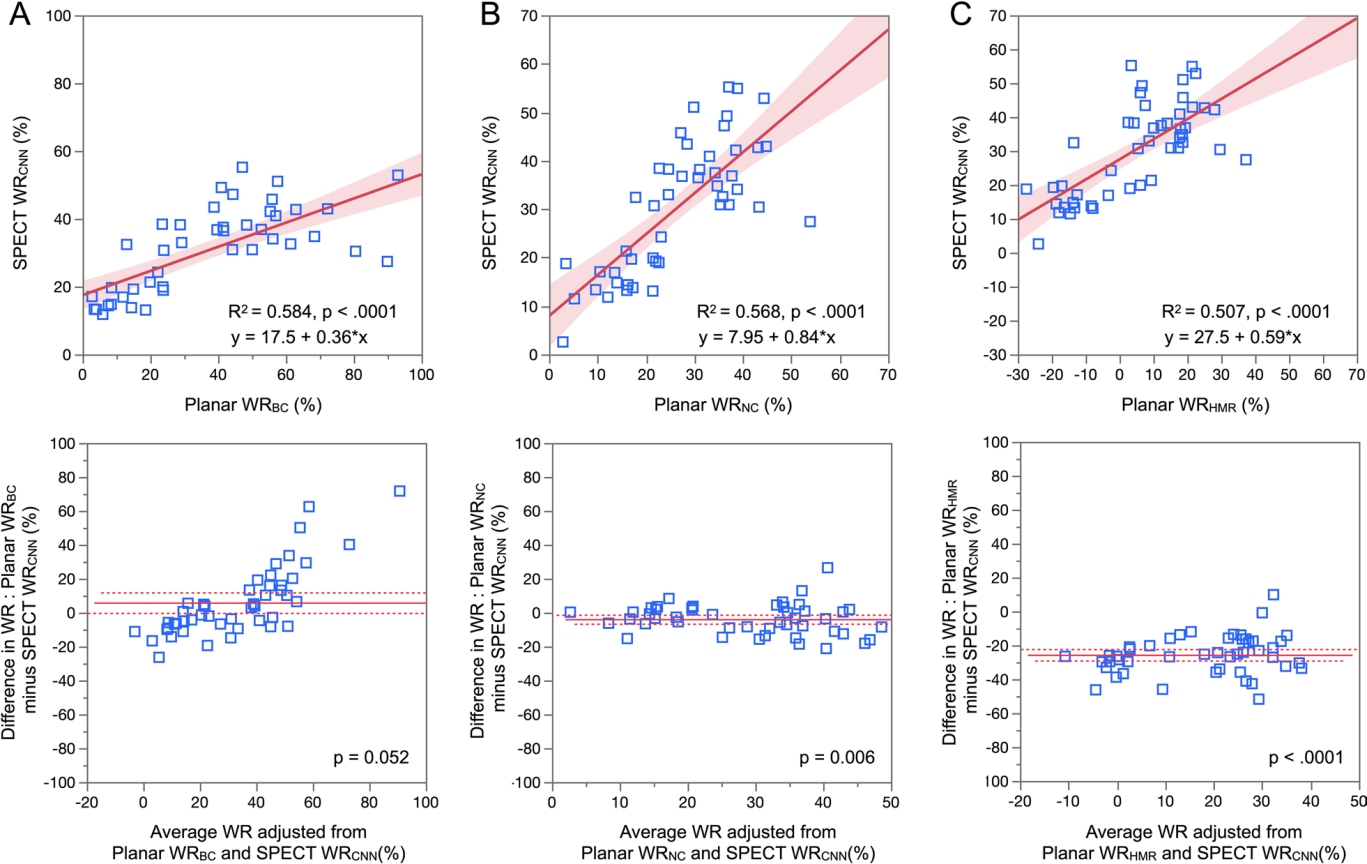
Relationships between washout rates calculated from SPECT images using CNN and planar images using conventional methods: linear regression lines (upper panels) and Bland–Altman plots (lower panels). SPECT WR vs. planar WR_BC_ (**A**), planar WR_NC_ (**B**), and planar WR_HMR_ (**C**). Shaded area, confidence of fit; dotted lines, 95% confidence intervals

We compared washout rates in SPECT images determined using CNN and in planar images determined using the conventional method. Correlations were significant between SPECT WR_CNN_ and planar WR parameters (*R*^2^ = 0.584, 0.568 and 0.507, *p* < 0.0001; Fig. 4). The systematic error between SPECT WR_CNN_ and planar WR_BC_ was on the borderline of significance as shown in Bland–Altman plots (*p* = 0.052). The SPECT WR_CNN_ showed systematically higher values compared with planar WR_NC_ and planar WR_HMR_ (*p* = 0.006 and *p* < 0.0001, respectively). The cutoff value of SPECT WR_CNN_ determined by linear regression with the upper limit of the normal range (34%) by the planar WR [24], was 30%. We assigned the patients to groups with normal and abnormal WR based on these cutoff values of SPECT WR_CNN_ and planar WR parameters (Table 2). Although six outliers remained, agreement between SPECT WR_CNN_ and planar WR_BC_ was good at 41 (87.2%) of 47 (Table 2A). The agreement rates between SPECT WR_CNN_ and planar WR_NC_ and planar WR_HMR_ were 78.7% and 72.3%, respectively (Table 2B and C).

**Table 2:** Washout rates determined from SPECT and planar images using CNN-based and standard methods, respectively

	Planar WR_BC_		Total
	< 34%	≥ 34%	
(A)			
SPECT WR_CNN_			
≤ 30%	23 (48.9%)	1 (2.1%)	24 (51.1%)
> 30%	5 (10.6%)	18 (38.3%)	23 (48.9%)
Total	28 (59.6%)	19 (40.4%)	47

	Planar WR_NC_		Total
	< 30.1%	≥ 30.1%	
(B)			
SPECT WR_CNN_			
≤ 30%	18 (38.3%)	1 (2.1%)	19 (40.4%)
> 30%	9 (19.2%)	19 (40.4%)	28 (59.6%)
Total	27 (57.5%)	20 (42.5%)	47

	Planar WR_HMR_		Total
	< 14.2%	≥ 14.2%	
(C)			
SPECT WR_CNN_			
≤ 30%	18 (38.3%)	1 (2.1%)	19 (40.4%)
> 30%	12 (25.5%)	16 (34.0%)	28 (59.6%)
Total	30 (63.8%)	17 (36.2%)	47

*Planar WR*_*BC*_, planar washout rate with background correction; *Planar WR*_*HMR*_, planar washout rate heart-to-mediastinum ratio; *Planar WR*_*NC*_, planar washout rate without background correction; *SPECT WR*_*CNN*_, SPECT washout rate using CNN.

## Discussion

While 3D quantitation for sympathetic nerve imaging is potentially useful, the feasibility of artificial intelligence for ^123^I-MIBG studies has not been verified. Therefore, the present study aimed to achieve segmentation and accurate quantitative values using CNN. The CNN segmented organs in 3D and calculated heart counts even when cardiac accumulation was low. The method presented herein could serve as a good foundation for 3D quantitative assessments.

### Advantages of SPECT over planar image acquisition

Although sympathetic nervous activity associated with ^123^I-MIBG has usually been estimated using planar imaging, the usefulness of HMR for diagnosis and prognosis has been confirmed. However, since HMR is a crude parameter based simply on cardiac and mediastinal regions, the planar method has inherently limited objectivity. Since anatomical structures including the heart are three-dimensional, the data obtained from two-dimensional images cannot perfectly separate these structures. In contrast, the 3D approach is fundamentally more appropriate for evaluating actual myocardial activity because it avoids organ overlap, and the myocardial wall excluding the LV cavity can be identified. We compared the new approach using SPECT images with conventional planar quantitation, but we could not strictly define myocardial walls. Since perfusion studies with ^99m^Tc-labeled tracers and X-ray CT studies were not included in the protocol for this study, the whole heart was segmented by the CNN algorithm. Further development will be required to strictly segment the myocardial wall.

The SPECT approach is also feasible as more institutions now have cadmium-zinc telluride SPECT cameras. Solid-state SPECT is capable of 3D evaluation with high-resolution and sensitivity, image acquisition is rapid, and radiation exposure is low due to a low injected dose, whereas planar images are not readily available. Therefore, the determination of total tracer uptake in organs using 3D images is an essential step and might lead to improved objectivity and diagnostic accuracy.

### Comparison with literature

Chen et al. assessed global quantitation of cardiac uptake using ^123^I-MIBG SPECT [26]. They calculated the SPECT HMR using a ratio of mean counts between cardiac and mediastinal volumes of interest (VOI), determined on transaxial images, and then compared them with the planar HMR. However, defining heart VOI using the SPECT quantitation tool includes some manual procedures. The shape of the heart VOI is an oval that does not precisely reflect the contour of the heart under examination. Here, we did not use a predefined heart model but automatically segmented the location of the heart and measured counts using the CNN. Since the CNN was trained on manual organ segmentation, the heart VOI was determined in a naturally shaped heart. Although the shape of heart cannot be traced in patients with extremely low cardiac activity, the CNN-segmented heart was placed on the approximate location of the cardiac region, and the average counts would not have significantly differed from those determined using a manually traced heart region.

### Heart segmentation and quantitation

The most crucial issue with heart segmentation using only SPECT images is the prevalence of low cardiac uptake in early images. We used a two-step approach to overcome this. Registration and final segmentation can be achieved using different methods, but we believe the two-step approach makes the model more robust and ensures consistent heart volumes for the two images.

The accumulation of ^123^I-MIBG is usually high in scintigraphy of the liver and heart, and moderate in the lungs. Since the distribution profile of ^123^I-MIBG is similar regardless of camera types, the CNN constructed herein will probably be applicable to other vendors, but further study will be required for confirmation.

Automated segmentation failed for one of our patients due to high tracer retention in an expanding renal pelvis. Unusually high or low accumulation in other locations, for example, the renal pelvis, large liver defects, extraordinary anatomical structures, can result in segmentation error. Although we already confirmed useful segmentation methods in most situations, adjustments might be required to minimize the frequency of errors. Training models on patients with atypical distribution might also improve performance. That is, the results will become more stable when the CNN is trained more on the anatomical locations of organs, as well as variations including regions of high accumulation outside the liver and heart.

The correlation of heart counts between CNN-based SPECT and conventional planar images was good (*r*^2^ = 0.73–0.86), whereas the correlation between CNN-based WR and planar WR parameters was lower than the CNN-based SPECT and planar heart counts (*r*^2^ = 0.51–0.58). Since WR is calculated as the subtraction and ratio of small values in reduced myocardial ^123^I-MIBG uptake, fluctuations in quantitation might have occurred at the higher range of WR. This variation resulted in the lower correlations between the CNN-based WR and planar WR parameters compared with normal ^123^I-MIBG uptake. However, the patients were separated well into normal and abnormal groups according to cutoff values for CNN-based WR and planar WR parameters.

### *Future directions for *^*123*^*I-MIBG imaging*

Since the data obtained from this study are relative quantitation, an absolute quantitation method using CNN should be established. For example, the standardized uptake value (SUV) can be calculated if data can be acquired with SPECT-CT and appropriate reconstruction method. To obtain better segmentation, additional anatomical information incorporating X-ray computed tomography with SPECT might be useful. Thereafter, a new three-dimensional index for globally measuring the total amount of ^123^I-MIBG might be developed. Such a novel quantitative approach will improve the uncertainty of the conventional method regarding two-dimensional quantitation and could be the next step towards absolute quantitation using the CNN. Including data from different cameras and reconstruction methods in CNN training would also improve the accuracy of segmentation.

## Limitations

This study had some limitations. Since we included a relatively small patient cohort, further investigations of larger patient cohorts are needed to develop more accurate segmentation. This study included patients with cardiac and neurological diseases, and some of them have yet to be finally diagnosed and/or their prognoses have yet to be confirmed. Clinical ^123^I-MIBG innervation studies in Japan have included both neurological and cardiac diseases. The present study aimed to create a methodology for 3D heart segmentation and the quantitation of both types of diseases. Therefore, consecutive patients with various backgrounds were selected to ensure that the CNN methods are broadly applicable, although disease-specific analyses, final diagnoses, and prognoses could not be included. To create the CNN architecture, three patients with indistinguishable lungs and liver were not included because the method relies on visualizing the contours of organs. Poor segmentation in one patient was due to excessive accumulation at another location. Such circumstances might be addressed by fusing SPECT-CT imaging with novel CNN-based segmentation. However, since X-ray CT has not been routinely applied for sympathetic nerve imaging at our institution, modifications of the study protocol will be required for further investigation.

## Conclusions

The CNN can be trained to determine organ contours and to automatically calculate heart counts and washout rates in ^123^I-MIBG SPECT images. Average SPECT heart counts calculated by CNN significantly correlated with those determined by conventional quantitation of planar images in patients with cardiac and neurological diseases. Washout rates also significantly correlated between SPECT with CNN segmentation and planar parameters. Automatic quantitation with CNN might have excellent potential and provide a foundation for the development of an absolute quantitative method.

## Supplementary Information

**Additional file 1.** Relationship of heart counts in patients with reduced uptake between CNN and conventional methods.

## Data Availability

The image datasets generated and/or analyzed during the current study are not publicly distributed, which is not approved by the Ethics Committees at Kanazawa University, but can be available from the corresponding author on reasonable request.

## Abbreviations

CNN: Convolutional neural network
H_BC_: Average heart counts with background correction
H_CNN_: Average heart counts using CNN-based segmentation
HMR: Heart-to-mediastinum ratio
MIBG: Metaiodobenzylguanidine
SPECT: Single-photon emission computed tomography
WR: Washout rates
WR_BC_: Washout rates with background correction
WR_CNN_: Washout rates using CNN-based segmentation
WR_NC_: Washout rates without background correction
