# Supplementary material for: Convolutional neural network-based automatic heart segmentation and quantitation in 123I-metaiodobenzylguanidine SPECT imaging
Source: EJNMMI Res. 2021 Oct 12;11:105. doi: 10.1186/s13550-021-00847-x (PMC8511236; doi:10.1186/s13550-021-00847-x)
Supplement: Supplementary file 1 — Additional file 1. Relationship of heart counts in patients with reduced uptake between CNN and conventional methods. [file 13550_2021_847_MOESM1_ESM.pdf]

Additional file 1

Supporting data for https://doi.org/10.1186/s13550-021-00847-x (EJNMMIRes 2021)

Convolutional neural network-based automatic heart segmentation and quantitation in ^{123}I- metaiodobenzylguanidine SPECT imaging

Saito S, Nakajima K, Edenbrandt L, Enqvist O, Ulén J, Kinuya S.

Relationship of heart counts in patients with reduced uptake between CNN and conventional methods

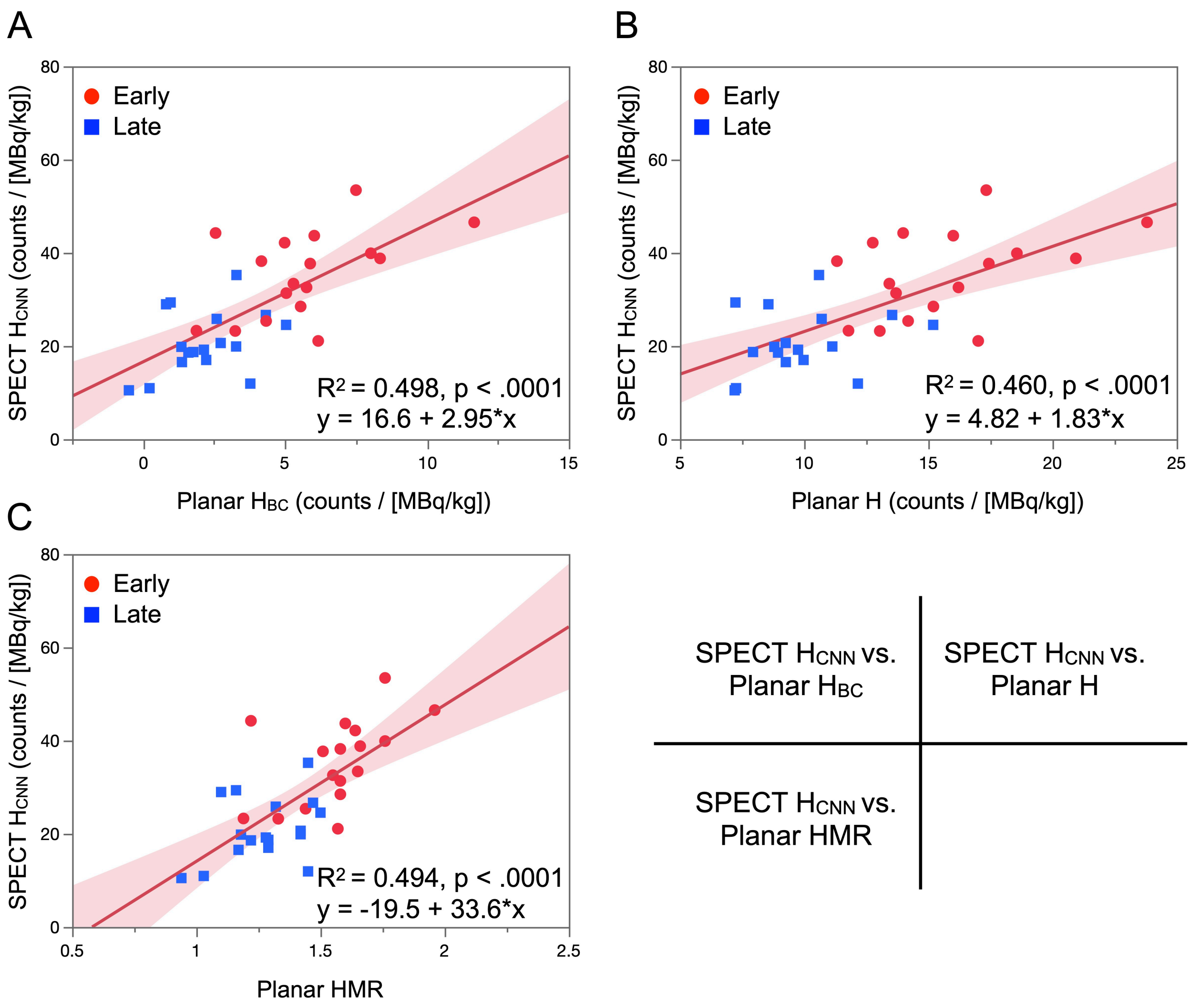

Figure S1. Relationships between average heart counts calculated from SPECT images using CNN and from conventional planar images in patients with reduced myocardial ^{123}I-MIBG uptake. SPECT H_{CNN} vs. Planar H_{BC} (A), Planar H (B), and Planar HMR (C). Red circles and blue squares, early and late images, respectively. Shaded area, confidence of fit.
